# RNA‐seq transcriptome analysis of a *Pseudomonas* strain with diversified catalytic properties growth under different culture medium

**DOI:** 10.1002/mbo3.357

**Published:** 2016-04-06

**Authors:** Jia‐Wei Yang, Dai‐Jun Zheng, Bao‐Dong Cui, Min Yang, Yong‐Zheng Chen

**Affiliations:** ^1^ Department of BiochemistryZunyi Medical UniversityZunyi563003China; ^2^ School of PharmacyZunyi Medical UniversityZunyi563000China

**Keywords:** Biocatalysis, differentially expressed gene, *Pseudomonas*, Transcriptome

## Abstract

Biocatalysis is an emerging strategy for the production of enantio‐pure organic molecules. However, lacking of commercially available enzymes restricts the widespread application of biocatalysis. In this study, we report a *Pseudomonas* strain which exhibited versatile oxidation activity to synthesize chiral sulfoxides when growing under M9‐toluene medium and reduction activity to synthesize chiral alcohols when on Luria‐Bertani (LB) medium, respectively. Further comparative transcriptome analysis on samples from these two cultural conditions has identified 1038 differentially expressed genes (DEG). Gene Ontology (GO) enrichment and KEGG pathways analysis demonstrate significant changes in protein synthesis, energy metabolism, and biosynthesis of metabolites when cells cultured under different conditions. We have identified eight candidate enzymes from this bacterial which may have the potential to be used for synthesis of chiral alcohol and sulfoxide chemicals. This work provides insights into the mechanism of diversity in catalytic properties of this *Pseudomonas* strain growth with different cultural conditions, as well as candidate enzymes for further biocatalysis of enantiomerically pure molecules and pharmaceuticals.

## Introduction

With the development of sustainable pharmaceutical preparation industry, green and environmental synthetic technologies have attracted great attentions. A low‐cost and green catalysis process is a more desirable way for the pharmaceutical synthesis as an alternative to traditional chemical catalysis (Huisman and Collier 2013). In recent years, the application of biocatalysts for the synthesis of valuable pharmaceutical intermediates and fine chemicals in industrial processes has become more common (Stryjewska et al. 2013). Compared to the traditional chemical catalysis, the biocatalytic process run with mild reaction conditions and decreased generation of waste or toxic by‐products (Alcalde et al. 2006; Woodley 2008). More importantly, the stereospecificity of enzymes offers an approach for producing enantio‐pure molecules, which means biocatalysis provide an effective synthetic strategy for the synthesis of chiral target chemicals with high enantioselectivity (Zhang et al. 2011). Specific reactions being replaced with biocatalysis have been applied in the pharmaceutical industry such as chiral amine and chiral alcohol synthesis (Desai 2011; Li et al. 2012; Aldridge 2013).

To perform efficient biocatalytic process, it is of great importance to discover the biocatalyst with high activity and enantioselectivity such as microbial communities or purified enzymes (Stryjewska et al. 2013). Over the past decade, lots of researchers have been reported in screening and applying whole‐cell microorganisms as biocatalysts for biotransformation (Fukuda et al. 2008; Schrewe et al. 2013; Zheng et al. 2014). With the development in molecular biology, screening and cloning genes to express recombinant enzyme proteins instead of natural microbial strains has become popular (Pollard and Woodley 2007; Lewis et al. 2011; Itoh et al. 2014). The expression level of recombinant enzymes was extremely increased compared to which in natural cells. Moreover, the properties of the recombinant enzyme could be further improved through protein engineering technology (Reetz 2011; Caswell et al. 2013; Alcalde 2015). These developments have facilitated the tailoring of enzyme properties and offering the possibility to create much more robust enzymes to meet specific reaction requirements for a wide range of synthetic and industrial applications (Zhang et al. 2011).

However, there are still many restrictions for widespread application of large‐scale biocatalysis in chiral pharmaceuticals, especially for the limited commercially available bioenzymes and the low yields of products (Zhang et al. 2011). In recent years, the application of genomic sequencing was been found to be greatly helpful for screening target genes encoding expected enzymes from a whole‐cell microorganism biocatalyst (Chen et al. 2014a; Frusciante et al. 2014; Li and Shao 2014). Nowadays, RNA‐seq, the next‐generation technique in sequencing of RNA, gives unprecedented detail about the transcriptional landscape of an organism (Young et al. 2010). It would become a powerful technology for discovering genes encoding expected bioenzymes. In our previous report, we have isolated a bacteria strain, named *Pseudomonas monteilii* CCTCC M2013683, from soil samples (Chen et al. 2014b). Our results showed that the whole‐cell biocatalyst could catalyze asymmetric oxidation of sulfides to chiral sulfoxides with high conversion (up to 99% yields) and enantioselectivity (up to 99% enantiomeric excess (*ee*)) when cultured with M9 medium using toluene as carbon source (M9‐toluene medium) (Chen et al. 2014b). Interestingly, this bacteria strain exhibited a distinct reduction activity to synthesis chiral alcohols when cultured with regular Luria‐Bertani (LB) medium in this study. As chiral sulfoxides and alcohols are key component in many pharmaceuticals such as fluoxetine, tomoxetine, and omeprazole (Feingersch et al. 2008; Wang et al. 2011), enzymes participated in these biocatalytic processes hold a great potential for applications in industrial catalysis. Thus, the comparative RNA‐seq transcriptome analysis was performed for further isolating and cloning genes encoding relevant enzymes involves in these biocatalytic processes.

## Materials and Methods

### Bacteria strains and cultivation

The *P. monteilii* CCTCC M2013683 strain isolated and identified in our laboratory was used for all experiments in this study. Bacteria cells grown in LB or M9‐agar plate (Zheng et al. 2014) were inoculated to 10 mL LB medium. After shaken at 300 rpm at 30°C for 8 h, 0.5 mL of bacterium suspensions was transferred into a 50 mL of liquid medium. The following cultures were incubated at 300 rpm at 30°C for another 16 h, and the cells were harvested by centrifugation. For cells cultured under M9‐toluene medium, 0.5 mL of bacterium cells was transferred into 50 mL of M9 liquid medium in a 250 mL shaking flask with ventilated plastic stopper. A quantity of 15 mL of plastic tube containing 0.5 mL toluene was put into the flask, and the vapor of toluene was used as carbon source (Zheng et al. 2014).

### Biocatalysis of *P. monteilii* CCTCC M2013683

Procedures of biocatalysis in this study were performed as previously reported (Chen et al. 2014b, b; Zheng et al. 2014). Cells were suspended in 5 mL of 50 mmol/L KH_2_PO_4_–K_2_HPO_4_ buffer (pH 7.0) to a density of 10 g dcw/L. A quantity of 10 mmol/L of substrate was added and the mixture was shaken at 300 rpm and 30^°^C for 24 h. All reaction mixture was extracted with 5 mL ethyl acetate containing 1 mmol/L benzyl alcohol as internal standard, and organic phase were analyzed by chiral HPLC. The ee values of products were determined using a Shi‐madzu^TM^ Prominence HPLC on a Daicel^TM^ OD‐H chiral column (250 × 4.6 mm, 5 *μ*m) at 25°C with a flow rate of 1 mL/min and UV detection at 220 nm, and the mobile phase used was hexane/isopropanol.

### RNA extraction, library preparation, and sequencing

Total RNA was extracted from about 10^8^ bacteria cells cultured in LB and M9 medium using the RNA isolation Kit (Tiangen, China), according to the manufacturer's instructions. RNA purity and concentration was checked using the NanoPhotometer^®^ spectrophotometer (IMPLEN, Munich, Germany) and Qubit^®^ RNA Assay Kit (Life Technologies, Carlsbad, CA, USA), respectively. The mRNA was purified from a total amount of 3 *μ*g RNA per sample and fragmented using divalent cations under elevated temperature. Random hexamer primer and M‐MuLV reverse transcriptase were used for cDNA first‐strand synthesis. Subsequently, second‐strand cDNA synthesis was performed using DNA polymerase I and RNase H. After adenylation of 3′ ends of cDNA fragments, NEB Next Adaptor with hairpin loop structure were ligated to prepare for hybridization. Then, the cDNA fragments were purified with AMPure XP system (Beckman Coulter, Beverly, CA, USA) to select fragments of 150~200 bp in length. PCR was then performed and products were purified (AMPure XP system) and library quality was assessed on the Agilent Bioanalyzer 2100 system. The sample clustering was performed on a cBot Cluster Generation System using TruSeq PE Cluster Kit v3‐cBot‐HS (Illumina, San Diego, CA, USA) according to the manufacturer's instructions. After cluster generation, the libraries were sequenced on an Illumina Hiseq 2000 platform and 100 bp paired‐end reads were generated.

### Reads mapping to the reference genome and quantification of gene expression level

Raw data (raw reads) were firstly processed through in‐house perl scripts and clean data (clean reads) were obtained by removing reads containing adapter or ploy‐N and low‐quality reads from raw data. Then, Q20, Q30, and GC content sequence of the clean data were calculated. Reference genome annotation files were downloaded from genome website (ftp://ftp.ncbi.nlm.nih.gov/genomes/Bacteria/Pseudomonas_putida_HB3267_uid184078/) directly. Bowtie2‐2.0.6 was used to build index of reference genome and align clean reads to reference genome (Langmead and Salzberg 2012). Mapping of clean reads to each gene was counted using HTSeq v0.5.4p3. And then RPKM (Reads Per Kilo base per Million mapped reads, Mortazavi et al. 2008) of each gene, was calculated based on the length of the gene and reads count mapped to this gene.

### Differential expression analysis

For each sequenced library, the read counts were adjusted by edger program package through one scaling normalized factor. Differential expression analysis of two conditions was performed using the DEGSeq R package (Wang et al. 2010). The *P*‐values were adjusted using the Benjamini & Hochberg method. Corrected *P*‐value of 0.005 and log_2_ (Fold change) of one were set as the threshold for significantly differential expression.

### GO and KEGG enrichment analysis of differentially expressed genes

Gene Ontology (GO) enrichment analysis of differentially expressed genes was implemented by the GO seq R package, in which gene length bias was corrected. GO terms with corrected *P‐*value less than 0.05 were considered significantly enriched by differential expressed genes. KOBAS software was used to test the statistical enrichment of differential expression genes in KEGG pathways (http://www.genome.jp/kegg/).

### Validation of differentially expressed genes by quantitative PCR

Reverse transcription‐quantitative PCR (RT‐qPCR) was performed to validate the DEGs from the RNA sequencing. Samples were tested using 2 × SYBR Green master mix (Transgen, Beijing, China) on the CFX96 Real‐Time System (BioRAD, Richmond, CA, USA) with three biological replicates. Relative expression was calculated using the delta‐delta‐Ct method with the 16S RNA as reference control. The reaction process was as follows: denaturation at 95°C for 3 min, followed by 40 cycles of amplification (95°C for 10sec and 60°C for 30sec). Primers for these genes were listed in Table S1.

## Results

### Catalytic activity of *P. monteilii* CCTCC M2013683 growing under different cultural conditions

**Figure 1:**
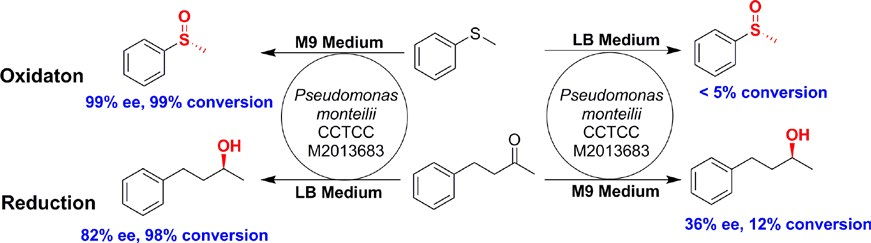
Schematic presentation of catalytic versatility *P. monteilii* CCTCC M2013683 growth under different culture medium. This bacterial strain showed oxidation activity growth under M9 medium (up) and reduction activity under LB medium (down).

In our previous report, a bacterial strain was isolated from soil samples and named *P. monteilii* CCTCC M2013683 (Chen et al. 2014b). When cultured with M9‐toluene medium, the whole‐cell bacteria could catalyze asymmetric oxidation of sulfides to chiral sulfoxides with 54–99% yields in 63–99% *ee* (Chen et al. 2014b). Moreover, the oxidation activity of this bacteria cell was activated when toluene was the only carbon and energy source (Chen et al. 2014b). When cultured under other carbon sources, like in LB medium with or without toluene, this bacterial strain exhibit less than 5% conversion (Fig. 1). Surprisingly, this whole‐cell biocatalyst can catalyze asymmetric reduction in 4‐phenylbutan‐2‐one to (*S*)‐4‐phenylbutan‐2‐ol with 97% conversion in 86% *ee* when cultivated in LB medium, while less than 12% conversion was detected when cultivated in other mediums like M9 medium with toluene or glucose (Fig. 1 and Fig. S1). These results illustrated that the bacterial cells expressed enzymes with different activities when growing in M9‐toluene versus in LB mediums, which implied a differential expression of genes encoding enzymes relevant to oxidation and reduction process. Thus, to further isolate and clone genes encoding relevant enzymes involves in these biocatalytic processes, transcriptomes of *P. monteilii* CCTCC M2013683 cultivated in M9‐toluene and LB mediums were sequenced and analyzed in our following study.

### RNA sequencing and aligning to the reference genome

Two cDNA libraries of *P. monteilii* CCTCC M2013683 cultured in LB medium or M9‐toulene medium were sequenced on an Illumina Hiseq 2000 platform and named as M2013683‐LB and M2013683‐M9, respectively. There were 26,870,378 and 27,504,392 raw reads generated from M2013683‐LB and M2013683‐M9 samples, respectively (Fig. S1, Table 1). Of the raw reads from M2013683‐LB, more than 94.55% bases has a *q* ≥ 20 (an error probability of 0.04%), and 95.09% for M2013683‐M9 (an error probability of 0.035%). A total of 26,691,664 clean reads of M2013683‐LB and 27,352,350 of M2013683‐M9 remained after filtration. The GC‐contents were 56.04% and 56.88% for M2013683‐LB and M2013683‐M9, respectively. The correlation value between M2013683‐LB and M2013683‐M9 was 0.817, which illustrated a high correlation between two samples.

**Table 1:** Quality of sequencing

Sample name	Raw reads	Clean reads	Clean bases	Error rate	Q20	Q30	GC content
M2013683‐LB‐1	13435189	13345832	1.67G	0.04%	95.53%	91.43%	56.71%
M2013683‐LB‐2	13435189	13345832	1.67G	0.04%	93.56%	88.37%	55.37%
M2013683‐M9‐1	13752196	13676175	1.71G	0.03%	96.02%	92.11%	57.47%
M2013683‐M9‐2	13752196	13676175	1.71G	0.04%	94.15%	89.22%	56.29%
M2013683‐LB‐total	26870378	26691664	3.34G	0.04%	94.55%	89.90%	56.04%
M2013683‐M9‐total	27504392	27352350	3.42G	0.035%	95.09%	90.67%	56.88%

M2013683‐LB‐1 and M2013683‐M9‐1 represent reads sequencing from the left; M2013683‐LB‐2 and M2013683‐M9‐2 represent reads sequencing from the right; Q20 and Q30 represent the percentage of bases with Phred value N20 and N30, respectively.

As the whole‐genome sequence of many *Pseudomonas* strains have been published, the sequence reads were then aligned to the database for further analysis of gene expression profiles. In order to choose the optimum reference genome database, the 16S‐rDNA of our *Pseudomonas* strain was amplified and submitted to GenBank (accession number: KU057954). The sequence of 16S‐rDNA was subjected to the BLAST (http://blast.ncbi.nlm.nih.gov/Blast.cgi) using nucleotide collection as database to get a phylogenetic tree (Fig. S2). The result illustrated that our strain is closest to *Pseudomonas putida* among all other species. After searching relevant reference genome information, the genome of *P. putida* HB3267 was used as the reference genome for the sequence reads alignment. The results showed that 92.44% of M2013683‐LB reads matched either to a unique (88.88%) or to multiple (3.56%) genomic locations, whereas 90.86% of M2013683‐M9 reads showed either a unique match (87.4%) or a multiple match (3.47%) (Table 2).

**Table 2:** Summary of reads aligned to the reference genome database

Sample name	LLA5	LTA5
Total reads	26691664	27352350
Total mapped	24672986 (92.44%)	24853670 (90.86%)
Multiple mapped	950419 (3.56%)	948032 (3.47%)
Uniquely mapped	23722567 (88.88%)	23905638 (87.4%)
Read‐1	11855732 (44.42%)	11948853 (43.68%)
Read‐2	11866835 (44.46%)	11956785 (43.71%)
Reads map to ‘+’	11862320 (44.44%)	11953586 (43.7%)
Reads map to ‘−’	11860247 (44.43%)	11952052 (43.7%)

### Global analysis of gene expression

**Figure 2:**
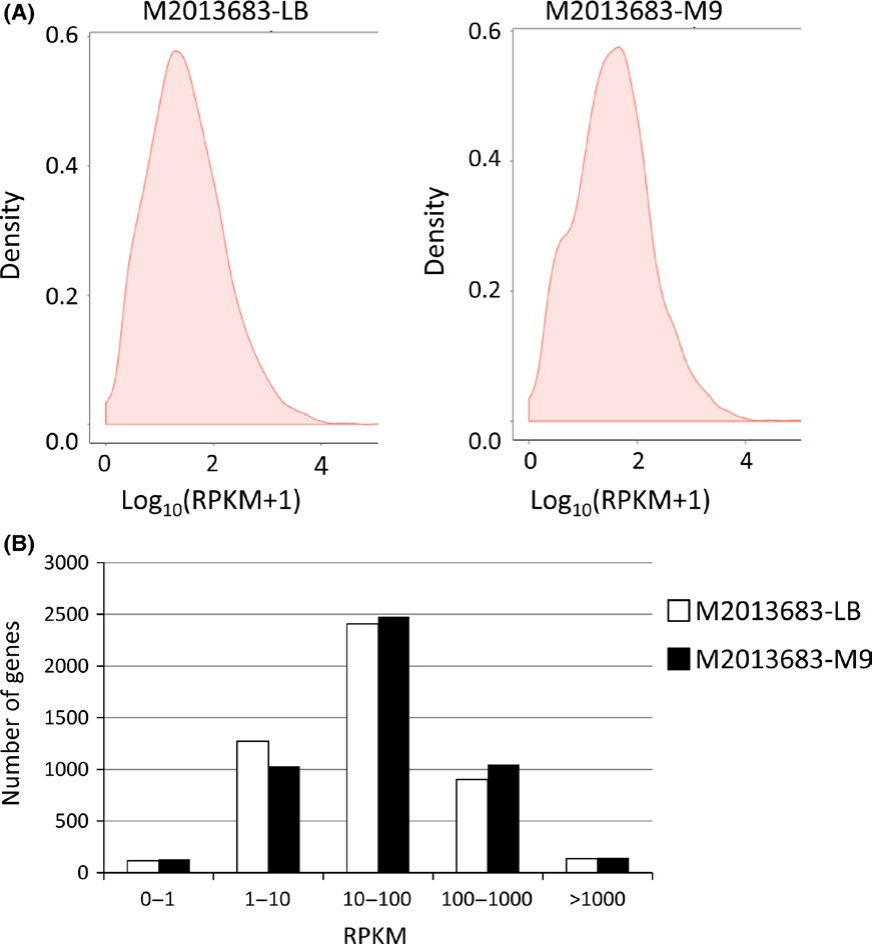
RPKM distribution density (A) and RPKM statistic (B) of M2013683‐LB and M2013683‐M9 samples.

After genome mapping, 4774 and 4776 genes were detected to be expressed in M2013683‐LB and M2013683‐M9, respectively. Among these genes, 4765 were commonly expressed between two groups. The RPKM value was used to evaluate the gene expression in two samples and showed no obvious difference in RPKM distribution density between M2013683‐LB and M2013683‐M9 samples (Fig. 2A). It was observed that there was the largest number of genes from both the M2013683‐LB and M2013683‐M9 groups when the RPKM value was from 10 to 100, while the fewest genes were identified when the RPKM value was from 0 to 1 (Fig. 2B). The RPKM value of majority differentially expressed genes between two samples was from 1 to 10 and from 10 to 100.

### Identification of differentially expressed genes between M2013683‐LB and M2013683‐M9

**Figure 3:**
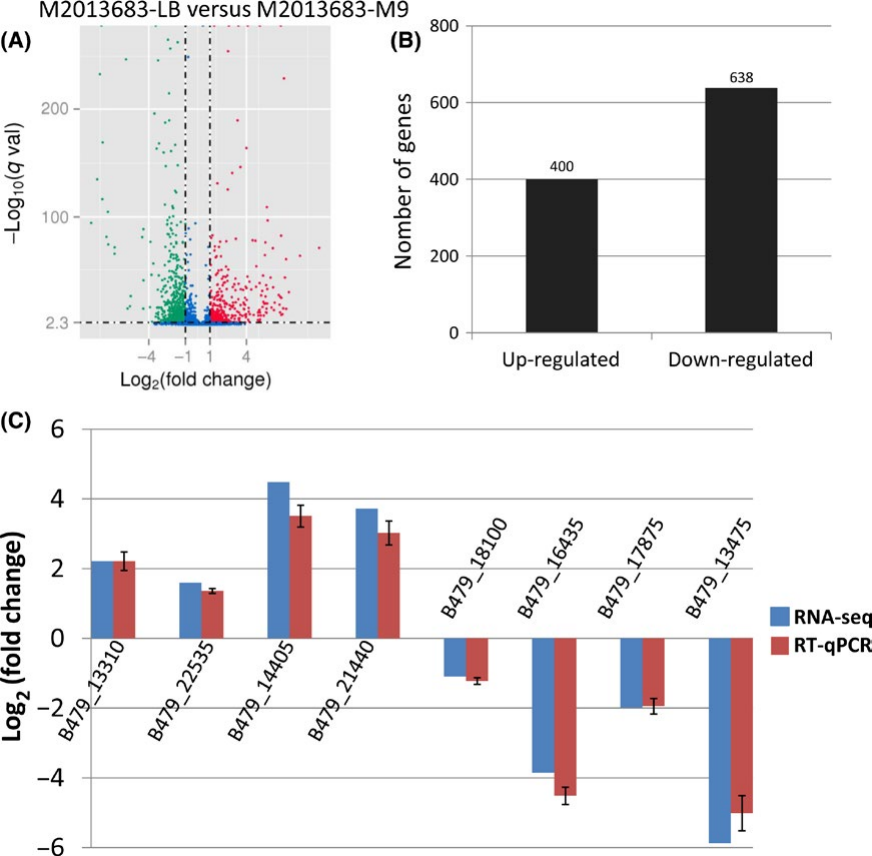
Comparison of differentially expressed genes identified between M2013683‐LB and M2013683‐M9. (A) Volcano Plots analysis of differentially expressed genes. The red dots represent differentially expressed genes (DEG)s up‐regulated in M2013683‐LB sample, the green dots represent DEGs down‐regulated in M2013683‐LB sample, and the blue dots represent not DEGs. (B) Quantitative statistic of differentially expressed genes. (C) Comparison of the expression level of the selected differently expressed genes as determined by RNA‐sequencing and RT‐qPCR

To better survey the biological mechanism of catalytic activity of M2013683‐LB and M2013683‐M9, it is important to identify the differentially expressed genes (DEG) between two samples. On the basis of the applied criteria (*q* < 0.005 and fold changes ≥2), 1038 genes (21.5% of all genes) were identified as significant DEGs between the two samples (Table S2). Of these, 400 DEGs (accounting for 38.5% of all significant DEGs) were up‐regulated while 638 DEGs (accounting for 61.5%) were down‐regulated in M2013683‐LB sample compared to M2013683‐M9 sample (Fig. 3A‐B, Table S2). The log_2_ (fold changes) were from 1 to 9.9436.

To further evaluate the data quality, eight DEGs of which four were up‐regulated and four were down‐regulated in M2013683‐LB were randomly selected for qRT‐PCR verification. The genes B479_13310, B479_22535, B479_14405, and B479_21440 which up‐regulated 2.2157, 1.6008, 4.4875, and 3.7257 log_2_‐fold in RNA‐sequencing data, showed 2.2155, 1.3599, 3.5096, and 3.0219 log_2_‐fold changes in the qRT‐PCR analyses, respectively. Likewise, in the RNA‐sequencing data, genes B479_18100, B479_16435, B479_17875, and B479_13475, which down‐regulated 1.0959, 3.8574, 1.9848, and 5.8745 log_2_ (fold change), showed 1.2230, 4.5150, 1.9460, and 5.0159 log_2_‐fold changes in the qRT‐PCR analyses, respectively (Fig. 3C). All eight genes showed similar expression patterns in both the qRT‐PCR and RNA‐sequencing analyses.

### GO enrichment analysis of DEGs

**Figure 4:**
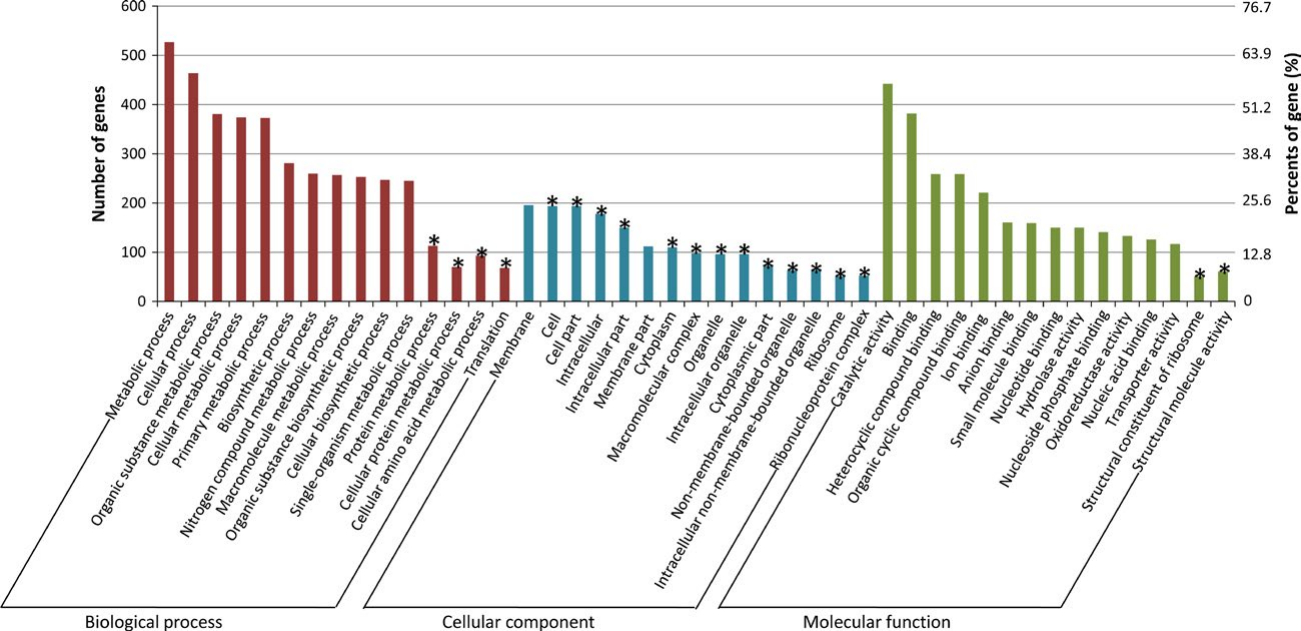
Functional categorization of differentially expressed genes in gene ontology (GO). The results are summarized in three main categories: biological process, molecular function, and cellular component, and 15 terms of each were exhibited. The asterisks indicates GO term significantly enriched.

Gene ontology (GO) enrichment is commonly used for describing the biological roles of genes and their products (Young et al. 2010). To determine the functions of differentially expressed genes, all DEGs were mapped to terms in the GO database and compared to the whole transcriptome background. Based on the GO categories, 782 of 1038 DEGs have a GO ID and could be categorized into 1760 functional groups in three main categories: biological process, cellular component, and molecular function (Table S3). In each of the three main categories of the GO classification, “metabolic process (GO: 0008152, 527 DEGs)”, “membrane (GO: 0016020, 196 DEGs)” and “catalytic activity (GO: 0003824, 442 DEGs)” terms were dominant (Fig. 4). And then, GO terms with corrected *P*‐value less than 0.05 were considered significantly enriched. The results showed four GO terms that were significantly enriched in the “biological process” category, 13 terms that were significantly enriched in the “cellular component,” and two terms in the “molecular function” category (Fig. 4). Interestingly, the most significantly enriched GO terms in each category was “translation (GO: 0006412)”, “ribosome (GO: 0005840),” and “structural constituent of ribosome (GO: 0003735)” (Table S3), which all related to proteins synthesis. These results suggested that synthesis of proteins in the bacteria was remarkably altered with the change of the culture conditions. In addition, 133 DEGs were categorized into “oxidoreductase activity (GO: 0016491)” group, including 37 up‐regulated and 96 down‐regulated genes in sample M2013683‐LB (Table S4). As the bacterial cells exhibited oxidation or reduction catalytic activities under different culture medium, some of the DEGs in “oxidoreductase activity” group would possibly related to these biocatalytic processes.

### KEGG pathway analysis of DEGs

**Figure 5:**
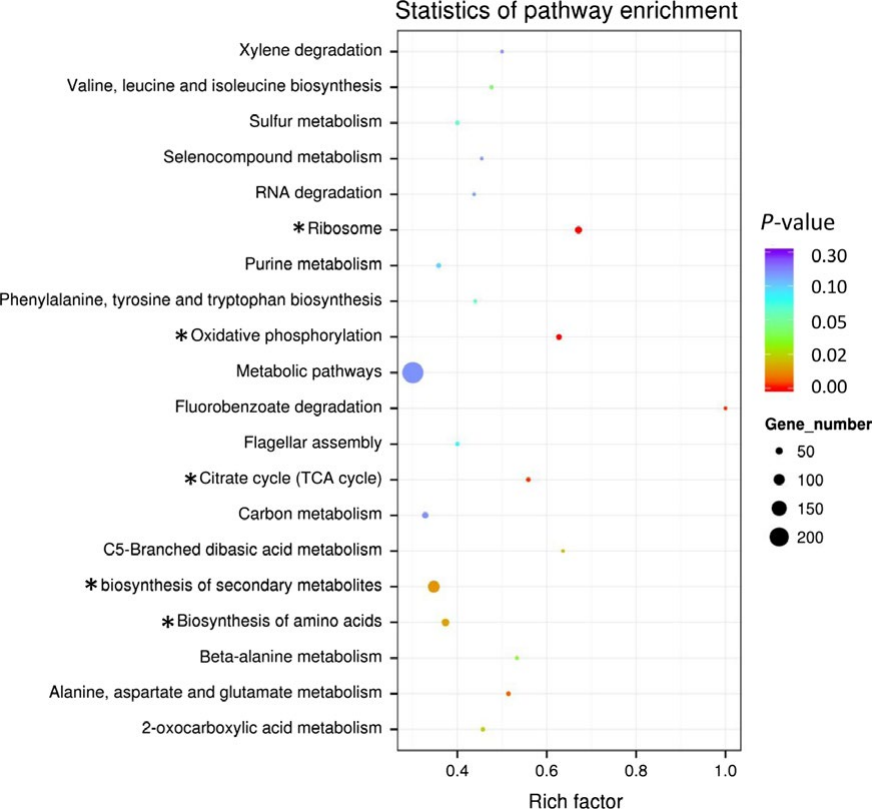
Scatterplot of differentially expressed genes enriched KEGG pathway. Rich factor represents the ratio of the number of differentially expressed genes (DEG)s and the number of all genes in the pathway. Top five pathways significantly enriched were marked with asterisks.

The KEGG pathway database is a widely accepted source for molecular pathway maps. Mapping of the DEGs to the KEGG pathways may provide an idea about the functional relevance of the gene lists corresponding to the high‐throughput expression data (Manyam et al. 2015). Thus, these 1038 DEGs were mapped to the terms in the KEGG database and categorized into 87 pathways with 11 of pathways were significantly enriched (*P* <0.05) (Table S5). Notably, the top five pathways that were significantly enriched with DEGs were “biosynthesis of secondary metabolites (ppuh01110, 108 DEGs)”, “biosynthesis of amino acids (ppuh01230, 56 DEGs),” “ribosome (ppuh03010, 51 DEGs),” “oxidative phosphorylation (ppuh00190, 32 DEGs)” and “Citrate cycle (ppuh00020, 19 DEGs)” (Fig. 5). Moreover, nine DEGs were enriched in “aromatic compounds degradation (ppuh01220)” pathway, including genes acting on aromatic ring conversion and cleavage, Beayer‐Villiger oxidation, dealkylation and monooxygenase reactions, etc. Among these nine DEGs, eight DEGs were up‐regulated in sample M2013683‐M9, which highly identity with that *P. monteilii* CCTCC M2013683 cells using toluene as the only carbon and energy source when cultured in M9 medium.

### Candidate DEGs involves in toluene degradation

**Figure 6:**
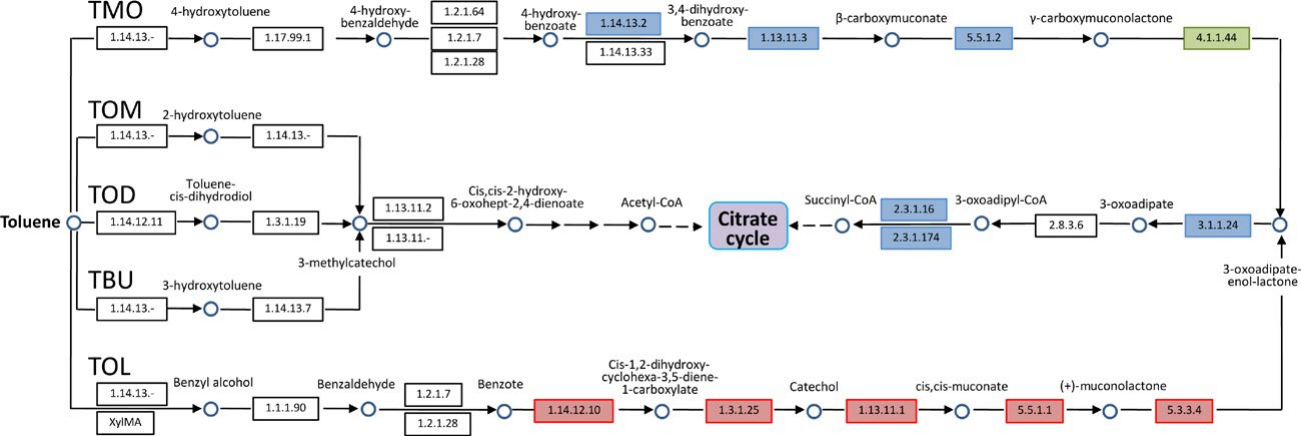
Putative toluene degradation pathways in aerobic bacteria constructed based on the KEGG database and the reference of Cao et al. 2009. The significantly up‐regulated and down‐regulated genes in M2013683‐M9 were labeled with red and green, respectively. The blue color represents genes with no expression differences between two samples, and the white color indicates genes that were not identified in the expression profile analysis.

Simple aromatic hydrocarbons, like benzene, toluene, ethylbenzene, and xylene have been widely used as carbon and energy sources for bacteria. Extensive researches have reported the biodegradation of these monoaromatic compounds (Cao et al. 2009). Five different biochemical pathways (TOD, TOM, TBU, TMO, TOL) have been characterized for toluene degradation by aerobic bacteria (Gulensory and Alvarez 1999; Cao et al. 2009). In this study, the toluene was the only carbon and energy source for cells cultured on M9‐toluene medium. Thus, we further analyzed the toluene degradation pathway in our bacterial cells based on the transcriptome sequencing data. The result revealed that no genes involved in TOD, TOM, and TBU pathways was detected to be expressed both in M2013683‐LB and M2013683‐M9 samples based on the KEGG pathway enrichment results (Fig. 6). Similarly, no TMO pathway involved genes were identified to be up‐regulated in M2013683‐M9 sample (Fig. 6). These results demonstrated that the toluene was not utilized through these pathways above in our bacterial cells. Conversely, there were 11 expressed genes were identified to involved in the TOL pathway, seven of which were differentially expressed between the M2013683‐LB and M2013683‐M9 samples. More importantly, all these seven DEGs showed obviously up‐regulated in M2013683‐M9 sample (Fig. 6 and Table S6), indicating that toluene could induce the expression of genes in TOL pathway. These results illustrated that our *Pseudomonas* strain probably utilize toluene as carbon source through the TOL pathway when gown on M9‐toluene medium, though other genes involved in this pathway were not identified since the annotation of reference genome was possibly imperfect.

### Candidate genes involved in biocatalysis of chiral sulfoxides and chiral alcohols

Based on our researches, the *P. monteilii* CCTCC M2013683 cells can synthesize chiral sulfoxides cultured only on M9‐toluene medium. Thus, seven DEGs in TOL pathway were first performed to analyze whether they were related to chiral sulfoxides synthesis. The result revealed that the toluate 1,2‐dioxygenase have the potential activity for chiral sulfoxides synthesis based on previous report (Boyd et al. 2012). Besides, 96 up‐regulated genes in sample M2013683‐M9 categorized into “oxidoreductase activity” group (GO: 0016491) were further analyzed for KEGG pathway analysis. The result demonstrated another three genes with asymmetric monooxygenase activity, which could also contribute to the biocatalysis of chiral sulfoxides (Table 3). On the other hand, the *P. monteilii* CCTCC M2013683 cells showed asymmetric reduction to synthesize chiral alcohols when cultured under LB medium. In order to screen genes encoding these enzymes, 37 up‐regulated genes in sample M2013683‐LB categorized into “oxidoreductase activity” group were further performed for KEGG pathway analysis. The result exhibited three genes participated in reduction of carbonyl group in ketones (Table 3), which could probably be related to the synthesis of chiral alcohol in this study. Therefore, these genes could be chosen as candidates for further cloning and recombinant expression for biocatalysis of chiral sulfoxide and alcohol chemicals and pharmaceuticals.

**Table 3:** Candidate enzymes probably involved in chiral alcohols and sulfoxides biocatalysis

Gene ID	log_2_(Fold change) (LB/M9)	Description	Reactions (KEGG)
B479_00610	2.2835	glucose‐methanol‐choline oxidoreductase; choline dehydrogenase	R01025
B479_22665	1.4689	3‐hydroxyisobutyrate dehydrogenase	R02047; R05066
B479_17770	1.4395	glycerol‐3‐phosphate dehydrogenase	R00842; R00844
B479_13475B479_13470	−5.8745−7.4727	Toluate 1,2‐dioxygenase	R07188; R05621
——	—–	Toluene monooxygenase	R02550
B479_23600	−2.6638	precorrin‐3B synthase	R05217
B479_05135	−1.2746	histidinol dehydrogenase	R01158; R01163
B479_05435	−1.1393	inosine 5′‐monophosphate dehydrogenase	R01130; R08240

## Discussion

Biocatalysis belongs to the greenest technologies for the synthesis of chiral molecules due to exquisite regioselectivity and stereoselectivity in water under mild conditions (Tao and Xu 2009). As biocatalysts have been extensively studied and increasingly applied in the industrial production of bulk chemicals and pharmaceuticals, there is an increasing demand for new biocatalysts with improved properties for industrial applications (Guazzaroni et al. 2015). Microorganisms played a central role for industrial applications as the major source of biocatalysts (Fukuda et al. 2008; Li et al. 2009; Boyd et al. 2012; Guazzaroni et al. 2015). In this study, the *Pseudomonas* strain *P. monteilii* CCTCC M2013683 exhibited versatile oxidation activity to synthesize chiral sulfoxides when growing on M9‐toluene medium and reduction activity to synthesize chiral alcohols on LB medium, respectively. To further investigate mechanisms in catalytic diversity and screen relevant enzymes in these biocatalytic processes, comparative transcriptome analysis was performed between these two samples.

After RNA sequencing and data analysis, 1038 DEGs were identified between two samples. The enrichment of 196 DEGs in “membrane” term of GO categories demonstrated that the transport of nutrition was changed in different cultural conditions. Similarly, 527 DEGs enriched in “metabolic process” and 442 DEGs enriched in “catalytic activity” illustrated that the expression of enzymes involved in metabolism were significantly changed for the acclimatization of cultural condition. These findings also explained the reason that the bacterial cells could exhibit distinct catalytic activity when cultured in LB and M9‐toluene medium. Moreover, because different carbon sources were used for the biosynthesis of metabolites when bacterial cells cultured in different medium, KEGG pathways like “biosynthesis of secondary metabolites” and “biosynthesis of amino acids” were significantly enriched with DEGs. Similarly, pathways involved in energy metabolism like “oxidative phosphorylation” and “Citrate cycle” were also significantly altered due to the different energy sources. As a consequence, the altered expression enzymes in these metabolic processes resulted in the diversity of catalytic properties when cells cultured under different conditions.

Bacterial species in *Pseudomonas* genus have been extensively studied on the aerobic degradation of aromatic compounds (Cao et al. 2009). According to KEGG analysis of DEGs and previous reports (Segura et al., 2005; Dominguez‐Cuevas et al., 2006; Cao et al. 2009), we hypothesized that the TOL pathway was activated to utilize toluene as carbon source when *P. monteilii* CCTCC M2013683 was grown on M9‐toluene medium. Based on the fact that *P. monteilii* CCTCC M2013683 cells could synthesize chiral sulfoxides when using the toluene as the only carbon and energy source, we had investigated the relationship of enzymes between TOL pathway and chiral sulfoxides synthesis. Previous report (Boyd et al. 2012) has showed that toluate 1,2‐dioxygenase have the potential activity for chiral sulfoxides synthesis. Besides, the toluene monooxygenase (Table 3), which participated in the first step in TOL toluene degradation pathway (Dominguez‐Cuevas et al., 2006), has been reported to be capable of synthesizing aromatic chiral sulfoxides (Feingersch et al. 2008). Even though the gene encoding this enzyme was not identified due to the possibly incomplete in reference genome, techniques like homology cloning can be used for further toluene monooxygenase genes isolation. In addition, we have screened another three genes up‐regulated in sample M2013683‐M9 and three genes up‐regulated in sample M2013683‐LB. Both of these genes were chosen as candidates for biocatalysis of chiral sulfoxides and alcohols in this study.

## Conclusions

In this study, the bacterial strain of *P. monteilii* CCTCC M2013683 showed a reductase activity to synthesize chiral alcohol when cultured in LB medium rather than oxidation activity which cultured in M9‐toluene medium reported previously. The transcriptome profile and DEG analysis revealed that protein synthesis, energy metabolism, and biosynthesis of metabolites were significantly changed when cells growth under different carbon and energy sources. Subsequently, the altered expression enzymes in these metabolic processes resulted in the diversity of catalytic properties when cells cultured with different mediums. More importantly, eight candidate enzymes were supposed to be participated in biocatalysis of chiral alcohols and sulfoxides. Further studies on gene cloning and recombinant expression will be performed for verification and application to synthesize optically pure alcohol and sulfoxide chemicals and pharmaceuticals.

## Conflict of Interest

The authors declare no conflict of interest.

## Supporting information


**Figure S1.** HPLC chromatogram of chiral alcohol using *P. monteilii* CCTCC M2013683 as biocatalyst.
**Figure S2.** Filtration of raw reads.
**Figure S3.** The correlation value between samples M2013683‐LB and M2013683‐M9.
**Figure S4.** Phylogenetic tree of *P. monteilii* CCTCC M2013683. The arrow represents sequence of *P. monteilii* CCTCC M2013683 16S rDNA.

**Table S1**. Primers used for qRT‐PCR to validate the data of RNA sequencing

**Table S2.** Differentially expressed genes between two groups. The *q* < 0.005 and log2. Fold change ≥1 were used as the threshold to judge the significance of gene expression difference.

**Table S3**. Gene Ontology (GO) annotation of DGEs between two samples

**Table S4**. DEGs involved in oxidoreductase activity.

**Table S5**. Pathway enrichment analysis of DEGs between two samples.

**Table S6**. Seven DEGs involved in TOL pathway in toulene utilization.
