# Supplementary material for: RNA‐seq transcriptome analysis of a Pseudomonas strain with diversified catalytic properties growth under different culture medium
Source: Microbiologyopen. 2016 Apr 6;5(4):626–36. doi: 10.1002/mbo3.357 (PMC4985596; doi:10.1002/mbo3.357)
Supplement: Supplementary file 1 — Figure S1. HPLC chromatogram of chiral alcohol using P. monteilii CCTCC M2013683 as biocatalyst. Figure S2. Filtration of raw reads. Figure S3. The correlation value between samples M2013683‐LB and M2013683‐M9. Figure S4. Phylogenetic tree of P. monteilii CCTCC M2013683. The arrow represents sequence of P. monteilii CCTCC M2013683 16S rDNA. [file MBO3-5-626-s001.docx]

**Supplementary Figures**

**Supplemental Fig. 1.** HPLC chromatogram of chiral alcohol using *P. monteilii* CCTCC M2013683 as biocatalyst.


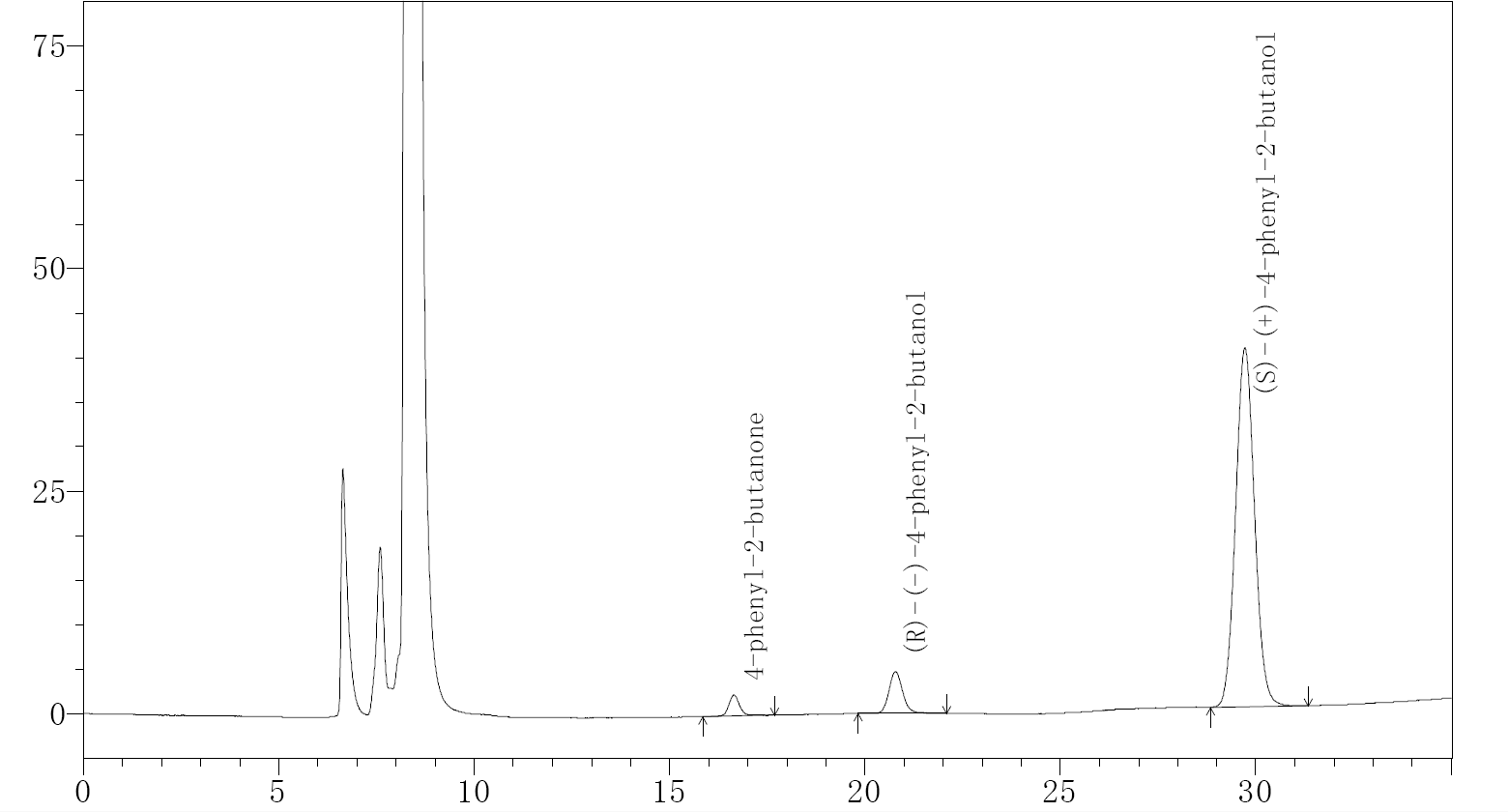


**Supplemental Fig. 2.** Filtration of raw reads


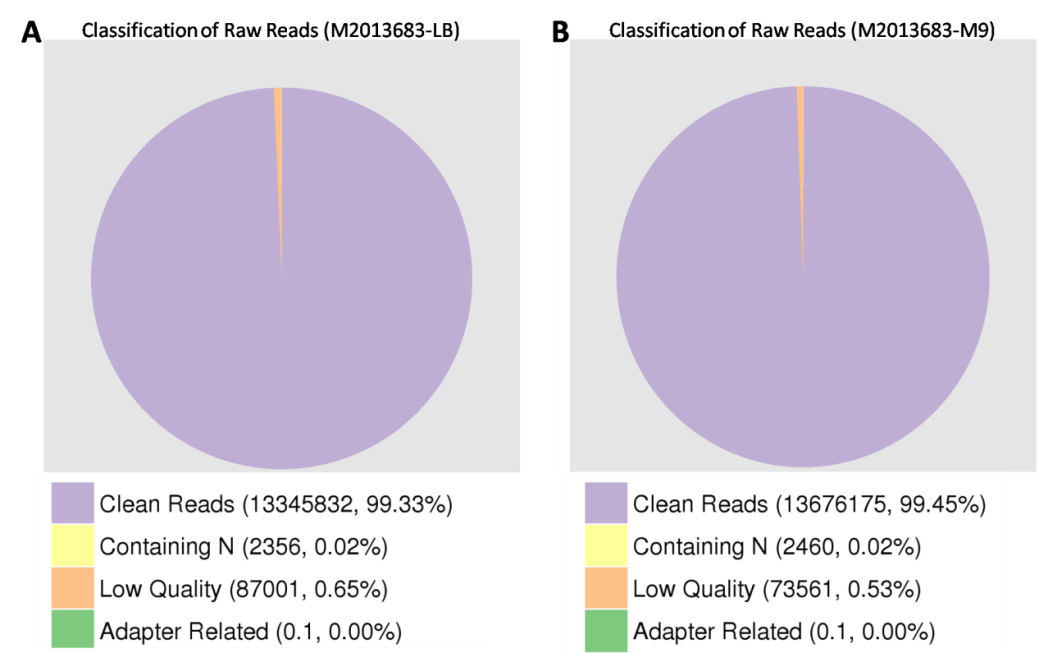


**Supplemental Fig. 3.** The correlation value between samples M2013683-LB and M2013683-M9


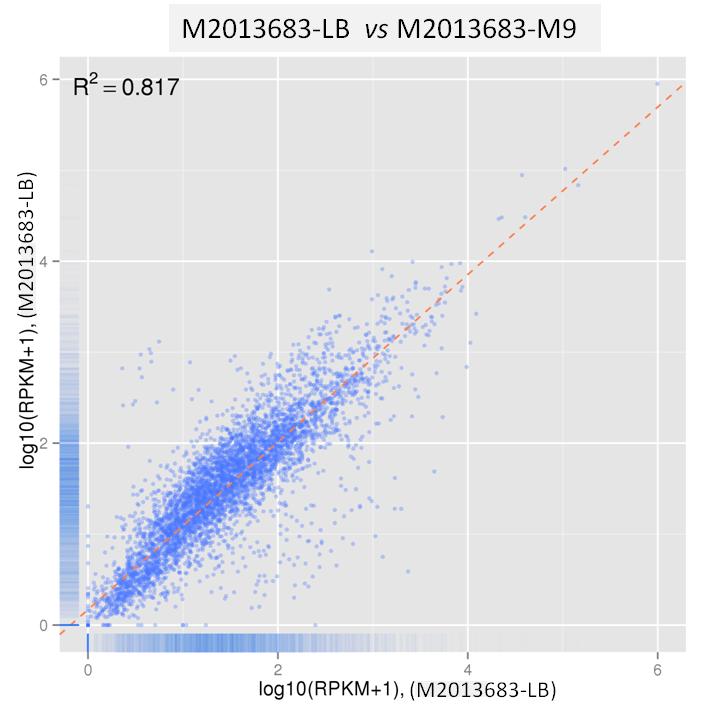


**Supplemental Fig. 4.** Phylogenetic tree of *P.* *monteilii* CCTCC M2013683. The arrow represents sequence of *P.* *monteilii* CCTCC M2013683 16S rDNA.
